# A Note on the Galvanomagnetic and Thermoelectric Coefficients of Tetragonal Crystalline Materials

**DOI:** 10.6028/jres.067A.031

**Published:** 1963-08-01

**Authors:** W. C. Hernandez, A. H. Kahn

## Abstract

The independent Hall, magnetoresistive, and thermoelectric coefficients for a tetragonal crystal have been tabulated and geometric configurations for their experimental measurement have been determined. These coefficients have been calculated on assumptions of several simple ellipsoidal models, in the range of nondegenerate statistics. Implications of experimentally observed isotropy or anisotropy of transport properties on the structure of the energy surfaces are noted.

## 1. Introduction

Detailed studies on the transport properties of rutile require tabulations of independent tensor coefficients. In this report we display these transport coefficients and method of measurement for galvanomagnetic and thermoelectric effects in tetragonal crystal structures. In anticipation of future experimental and theoretical results these transport coefficients have been calculated on the basis of various ellipsoidal energy band structures compatible with tetragonal symmetry.

## 2. Galvanomagnetic Coefficients

The electric current density components *J_i_* and electric field components *E_i_* are related by the conductivity tensor *σ_ij_* in the following way:

$$J_{i}=\sigma_{ij}(H)E_{j},$$ (1)

where the conductivity is understood to be a function of the magnetic field vector **H** and the Einstein summation convention is used. For weak field galvanomagnetic effects a series expansion of *σ* in powers of *H* up to second order is frequently sufficient. To second order the current is given by

$$J_{i}=\sigma_{ij}^{\left(0\right)}E_{j}+\sigma_{ijk}^{\left(1\right)}E_{j}H_{k}+\sigma_{ijkl}^{\left(2\right)}E_{j}H_{k}H_{l}.$$ (2)

The superscripts indicate the corresponding power of *H*, but these will frequently be omitted without causing ambiguity. The restrictions of crystal symmetry [1]^1^ and the Onsager reciprocal relations [2, 3] greatly limit the number of independent conductivity coefficients in eq (2). The symmetry conditions of the tetragonal point group of rutile (*D*_4_*_h_*) require that eq (2) remain invariant under rotations about three perpendicular 2-fold axes, one of which is also a 4-fold axis. The Onsager relations require that $\sigma_{ij}^{\left(0\right)}$ and $\sigma_{ijkl}^{\left(2\right)}$ be symmetric, while $\sigma_{ijk}^{\left(1\right)}$ must be antisymmetric on interchange of *i* and *j.* The resulting independent components are produced in tables 1, 2 and 3. Note that for zero order there are two coefficients, for first order two, and for second order there are seven.

More often than not, experimental measurements yield resistivity coefficients, the reciprocal of the conductivity. The resistivity coefficients are given up to the second order by

$$E_{i}=\left[\rho_{ij}^{\left(0\right)}+\rho_{ijk}^{\left(1\right)}H_{k}+\rho_{ijkl}^{\left(2\right)}H_{k}H_{l}\right]J_{j}.$$ (3)

The same symmetry conditions apply as before, and the independent resistive coefficients will be identified by the same subscripts. The coefficients are given in table 4.

## 3. Experimental Determination of Magnetoresistive Coefficients

The zero order resistivities are measured in the usual way. The two coefficients *ρ*_11_ and *ρ*_33_ refer to the resistivities perpendicular and parallel to the 4-fold axis, respectively. Typical experimental orientations and geometries have been determined for obtaining the magnetoresistive coefficients. The first order, or Hall, coefficients are described in table 5, and the second order in table 6. Numerals 1, 2, 3 in the tables refer to crystal axes; 1 and 2 being 2-fold and 3 being the 4-fold axis.

## 4. Magnetoconductive Coefficients on the Basis of Simple Ellipsoidal Models

Experimental and theoretical studies of band structure and transport coefficients may be aided by calculations of the transport properties of simple many ellipsoid energy surface configurations. This type of analysis of magnetoresistance has been very successful in the determination of the band structures of silicon and germanium [4, 5].

First we discuss the magnetoconductive coefficients of a single ellipsoid. Rather than using a Boltzmann equation, we follow Brooks [5] method of following the motion of a single charge carrier on the energy surface under the influence of applied fields, and then averaging over a Maxwell distribution of carriers. The equations of motion for an electron on an ellipsoidal energy surface, in the effective mass approximation, are the three equations

$$\frac{dv_{i}}{dt}=\frac{e}{m_{i}}E_{i}+\frac{e}{m_{i}c}\left(v_{j}H_{k}-v_{k}H_{j}\right),$$ (4)

where the subscripts indicate components or effective masses along principal directions of the ellipsoid, and *i, j, k* are cyclic permutations of 1, 2, 3. These equations of motion are solved to second order in **H**, giving the velocity as a function of time. The acquired motion is obtained by averaging over a distribution of free drift times given by (1/*τ*)*e^−t^*^/^*^τ^.* The resultant acquired current averaged over the Maxwell distribution of electrons is

$$\begin{array}{l} \langle ev_{i}\rangle =\frac{e^{2}\langle \tau \rangle }{m_{i}}E_{i}+\frac{e^{3}\langle \tau^{2}\rangle }{m_{i}m_{j}c}E_{j}H_{k}-\frac{e^{3}\langle \tau^{2}\rangle }{m_{i}m_{k}c}E_{k}H_{j}\\ \;-\frac{e^{4}\langle \tau^{3}\rangle }{m_{i}^{2}m_{k}c^{2}}E_{i}H_{j}^{2}-\frac{e^{4}\langle \tau^{3}\rangle }{m_{i}^{2}m_{j}c^{2}}E_{i}H_{k}^{2}\\ \;+\frac{e^{4}\langle \tau^{3}\rangle }{m_{i}m_{j}m_{k}c^{2}}E_{k}H_{i}H_{k}+\frac{e^{4}\langle \tau^{3}\rangle }{m_{i}m_{j}m_{k}c^{2}}E_{j}H_{j}H_{k}. \end{array}$$ (5)

In eq (5) it is understood that the relaxation time *τ* is independent of direction, but perhaps dependent on energy. The angular brackets indicate averages of *τ* over the Maxwellian energy distribution. The total current is just the average current multiplied by the number of electrons in the ellipsoid.

We shall consider now the resistivity coefficients for several configurations of ellipsoids compatible with crystal symmetry. The assumption is made that the total conductivity of the array is the sum of the conductivities of the individual ellipsoids. Thus we assume that a relaxation time exists for each ellipsoid, and that intervalley scattering is negligible.

### 4.1. Single Ellipsoid

**Figure 1:**
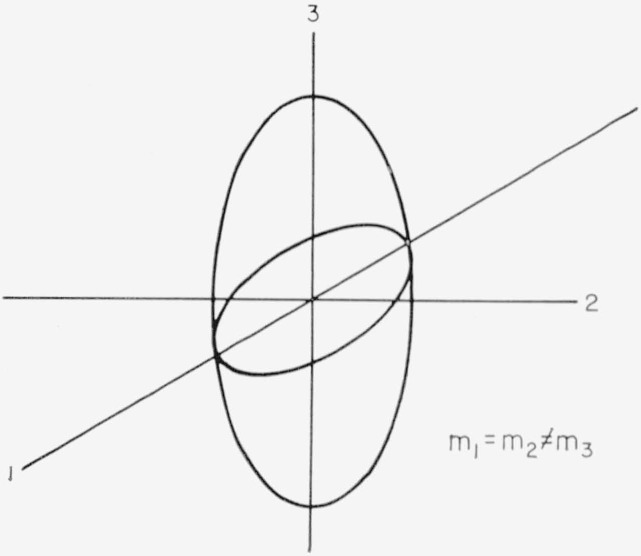
Single ellipsoid for tetragonal symmetry.

In this case *m*_1_=*m*_2_≠*m*_3_. The configuration is shown in figure 1. The results for the resistive coefficients are as follows:

$$\begin{array}{l} \rho_{123}=-\rho_{132}=\frac{-1}{nec}\frac{\langle \tau^{2}\rangle }{{\langle \tau \rangle }^{2}}\\ \rho_{1212}=\frac{m_{1}}{m_{3}}\rho_{1313}=\frac{1}{2m_{3}nc^{2}\langle \tau \rangle }\left[\frac{\langle \tau^{3}\rangle \langle \tau \rangle -{\langle \tau^{2}\rangle }^{2}}{{\langle \tau \rangle }^{2}}\right]\\ \rho_{1133}=\rho_{3311}=\frac{m_{3}}{m_{1}}\rho_{1122}=\frac{1}{m_{1}nc^{2}\langle \tau \rangle }\left[\frac{\langle \tau^{3}\rangle \langle \tau \rangle -{\langle \tau^{2}\rangle }^{2}}{{\langle \tau \rangle }^{2}}\right]\\ \rho_{1111}=\rho_{3333}=0. \end{array}$$

### 4.2. Four Ellipsoid Model

**Figure 2:**
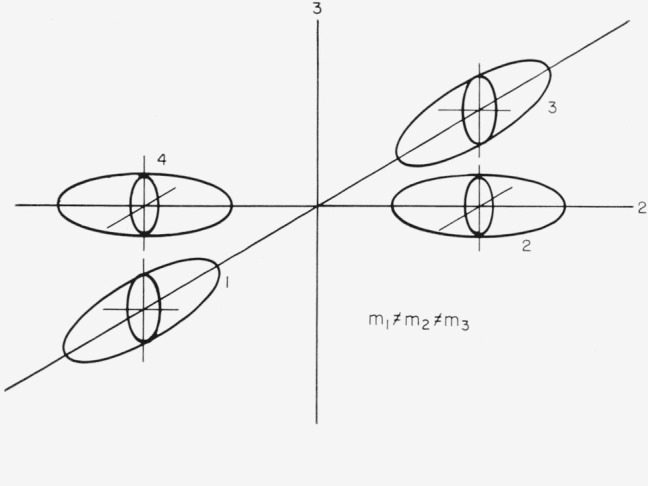
Four ellipsoids for tetragonal symmetry.

In this case the ellipsoids can have three different masses, but are placed along the 2-fold axes, as shown in figure 2. The effective masses *m*_1_, *m*_2_, *m*_3_ apply to ellipsoid number 1 of figure 2. The masses for the other ellipsoids are obtained by rotation. The resistive coefficients are:

$$\begin{array}{l} \rho_{123}=\frac{-1}{nec}\frac{\langle \tau^{2}\rangle }{{\langle \tau \rangle }^{2}}\frac{4m_{1}m_{2}}{{\left(m_{1}+m_{2}\right)}^{2}}\\ \rho_{132}=\frac{1}{nec}\frac{\langle \tau^{2}\rangle }{{\langle \tau \rangle }^{2}} \end{array}$$

Notice that $\frac{\rho_{123}}{\rho_{132}}=\frac{4m_{1}m_{2}}{{\left(m_{1}+m_{2}\right)}^{2}}=\text{constant}$.

$$\begin{array}{l} \rho_{1122}=\frac{1}{nm_{3}c^{2}\langle \tau \rangle }\left[\frac{2\left(m_{1}^{2}+m_{2}^{2}\right)}{{\left(m_{1}+m_{2}\right)}^{2}}\frac{\langle \tau^{3}\rangle \langle \tau \rangle }{{\langle \tau \rangle }^{2}}-\frac{{\langle \tau^{2}\rangle }^{2}}{{\langle \tau \rangle }^{2}}\right]\\ \rho_{1212}=\frac{-1}{2nm_{3}c^{2}\langle \tau \rangle }\left[\frac{4m_{1}m_{2}}{{\left(m_{1}+m_{2}\right)}^{2}}\langle \tau^{3}\rangle \langle \tau \rangle -\frac{{\langle \tau^{2}\rangle }^{2}}{{\langle \tau \rangle }^{2}}\right]\\ \rho_{1133}=\frac{2}{\left(m_{1}+m_{2}\right)mc^{2}\langle \tau \rangle }\left[\frac{\langle \tau^{3}\rangle \langle \tau \rangle }{{\langle \tau \rangle }^{2}}-\frac{4m_{1}m_{2}}{{\left(m_{1}+m_{2}\right)}^{2}}\frac{{\langle \tau^{2}\rangle }^{2}}{{\langle \tau \rangle }^{2}}\right]\\ \rho_{1313}=\frac{-1}{\left(m_{1}+m_{2}\right)nc^{2}\langle \tau \rangle }\left[\frac{\langle \tau \rangle \langle \tau^{3}\rangle -{\langle \tau^{2}\rangle }^{2}}{{\langle \tau \rangle }^{2}}\right]\\ \rho_{3311}=\frac{\left(m_{1}+m_{2}\right)}{2m_{1}m_{2}nc^{2}\langle \tau \rangle }\left[\frac{\langle \tau \rangle \langle \tau^{3}\rangle -{\langle \tau^{2}\rangle }^{2}}{{\langle \tau \rangle }^{2}}\right]\\ \rho_{1111}=\rho_{3333}=0. \end{array}$$

### 4.3. Five Ellipsoids

**Figure 3:**
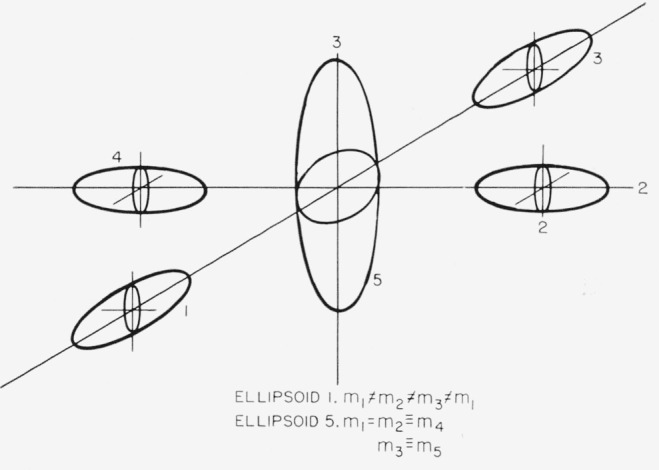
Five ellipsoids for tetragonal symmetry.

There is a possibility that more than one energy surface will be populated. To give an example, we shall consider a combination of cases (a) and (b), as shown in figure 3. The resultant resistivities are:

$$\begin{array}{l} \rho_{123}=\frac{-1}{ec}\frac{\langle \tau^{2}\rangle }{{\langle \tau \rangle }^{2}}\frac{m_{1}m_{2}m_{4}^{2}n_{1}+m_{1}^{2}m_{2}^{2}n_{2}}{{\left[\left(m_{1}+m_{2}\right)m_{4}\frac{n_{1}}{2}+m_{1}m_{2}n_{2}\right]}^{2}}\\ \rho_{132}=\frac{1}{ec}\frac{\langle \tau^{2}\rangle }{\langle \tau \rangle }\frac{\left[\left(m_{1}+m_{2}\right)m_{4}m_{5}\frac{n_{1}}{2}+m_{1}m_{2}m_{3}n_{2}\right]}{\left(m_{5}n_{1}+m_{3}n_{2}\right)\left[\left(m_{1}+m_{2}\right)m_{4}\frac{n_{1}}{2}+m_{1}m_{2}n_{2}\right]}. \end{array}$$

$$\begin{array}{l} \rho_{1212}=1\frac{1}{c^{2}}\frac{\langle \tau^{3}\rangle }{{\langle \tau \rangle }^{2}}\frac{\left[m_{4}^{2}m_{5}n_{1}+m_{1}m_{2}m_{3}n_{2}\right]m_{1}m_{2}}{{\left[\left(m_{1}+m_{2}\right)m_{4}\frac{n_{1}}{2}+m_{1}m_{2}n_{2}\right]}^{2}m_{3}m_{5}}+\frac{1}{2c^{2}}\frac{{\langle \tau^{2}\rangle }^{2}}{{\langle \tau \rangle }^{3}}\frac{{\left[\left(m_{1}+m_{2}\right)m_{4}m_{5}\frac{n_{1}}{2}+m_{1}m_{2}m_{3}n_{2}\right]}^{2}}{{\left[\left(m_{1}+m_{2}\right)m_{4}\frac{n_{1}}{2}+m_{1}m_{2}n_{2}\right]}^{2}\left[m_{5}n_{1}+m_{3}n_{2}\right]m_{3}m_{5}}\\ \rho_{1122}=\frac{1}{c^{2}}\frac{\langle \tau^{3}\rangle }{{\langle \tau \rangle }^{2}}\frac{\left[\left(m_{1}^{2}+m_{2}^{2}\right)m_{4}^{2}m_{5}\frac{n_{1}}{2}+m_{1}^{2}m_{2}^{2}m_{3}n_{2}\right]}{{\left[\left(m_{1}+m_{2}\right)m_{4}\frac{n_{1}}{2}+m_{1}m_{2}n_{2}\right]}^{2}m_{3}m_{5}}-\frac{1}{2c^{2}}\frac{{\langle \tau^{2}\rangle }^{2}}{{\langle \tau \rangle }^{3}}\frac{{\left[\left(m_{1}+m_{2}\right)m_{4}m_{5}\frac{n_{1}}{2}+m_{1}m_{2}m_{3}n_{2}\right]}^{2}}{{\left[\left(m_{1}+m_{2}\right)m_{4}\frac{n_{1}}{2}+m_{1}m_{2}n_{2}\right]}^{2}\left[m_{5}n_{1}+m_{3}n_{2}\right]m_{3}m_{5}}\\ \rho_{1133}=\frac{1}{c^{2}}\frac{\langle \tau^{3}\rangle }{{\langle \tau \rangle }^{2}}\frac{\left[\left(m_{1}+m_{2}\right)m_{4}^{3}\frac{n_{1}}{2}+m_{1}^{2}m_{2}^{2}n_{2}\right]}{{\left[\left(m_{1}+m_{2}\right)m_{4}\frac{n_{1}}{2}+m_{1}m_{2}n_{2}\right]}^{2}m_{4}}-\frac{1}{c^{2}}\frac{{\langle \tau^{2}\rangle }^{2}}{{\langle \tau \rangle }^{3}}\frac{{\left[m_{4}^{2}n_{1}+m_{1}m_{2}n_{2}\right]}^{2}m_{1}m_{2}}{{\left[\left(m_{1}+m_{2}\right)m_{4}\frac{n_{1}}{2}+m_{1}m_{2}n_{2}\right]}^{3}m_{4}}\\ \rho_{1313}=\frac{-1}{c^{2}}\frac{\langle \tau^{3}\rangle }{{\langle \tau \rangle }^{2}}\frac{\left[m_{4}^{2}m_{5}n_{1}+m_{1}m_{2}m_{3}n_{2}\right]}{\left[\left(m_{1}+m_{2}\right)m_{4}\frac{n_{1}}{2}+m_{1}m_{2}n_{2}\right]\left[m_{5}n_{1}+m_{3}n_{2}\right]m_{4}}\\ +\frac{1}{2c^{2}}\frac{{\langle \tau^{2}\rangle }^{2}}{{\langle \tau \rangle }^{3}}\frac{\left[m_{4}^{2}n_{1}+m_{1}m_{2}n_{2}\right]\;\left[\left(m_{1}+m_{2}\right)m_{4}m_{5}\frac{n_{1}}{2}+m_{1}m_{2}m_{3}n_{2}\right]}{{\left[\left(m_{1}+m_{2}\right)m_{4}\frac{n_{1}}{2}+m_{1}m_{2}n_{2}\right]}^{2}\left[m_{5}n_{1}+m_{3}n_{2}\right]m_{4}}\\ \rho_{3311}=\frac{1}{c^{2}}\frac{\langle \tau^{3}\rangle }{{\langle \tau \rangle }^{2}}\frac{\left[\left(m_{1}+m_{2}\right)m_{4}^{2}m_{5}\frac{n_{1}}{2}+m_{1}m_{2}m_{3}^{2}n_{2}\right]}{{\left[m_{5}n_{1}+m_{3}n_{2}\right]}^{2}m_{1}m_{2}m_{4}}-\frac{1}{c^{2}}\frac{{\langle \tau^{2}\rangle }^{2}}{{\langle \tau \rangle }^{3}}\frac{{\left[\left(m_{1}+m_{2}\right)m_{4}m_{5}\frac{n_{1}}{2}+m_{1}m_{2}m_{3}n_{2}\right]}^{2}}{\left[\left(m_{1}+m_{2}\right)m_{4}\frac{n_{1}}{2}+m_{1}m_{2}n_{2}\right]{\left[m_{5}n_{1}+m_{3}n_{2}\right]}^{2}m_{1}m_{2}m_{4}}. \end{array}$$

In all the previous formulas the temperature dependence of the resistivities depends upon averages of several powers of *τ*. For power law scattering, *τ=τ*_0_*ϵ^−p^*, where *ϵ* is the energy and *p* the power, we have

$$\langle \tau^{n}\rangle =\tau_{0}^{n}\frac{{\left(kT\right)}^{-np}\Gamma \left(\frac{5}{2}-np\right)}{\Gamma \left(\frac{5}{2}\right)}.$$ (6)

It might be worth noting that in tetragonal crystals anisotropic effects make themselves known in lower order magnetoresistive coefficients than for cubic cases. Thus, for example, information concerning anisotropy of the energy surfaces can be obtained from the Hall coefficient of TiO_2_, while one must go to magnetoresistive to see the lowest order effects of cubic anisotropy in Si or Ge.

## 5. Thermoelectric Power

The electric current density components *J_i_* and the thermal current components *Q_i_* are related to the electric field components *E_i_* and the temperature gradient components $\frac{\partial T}{\partial X_{j}}$ in the following way:

$$J_{i}=A_{ij}^{11}E_{j}+A_{ij}^{12}\frac{\partial T}{\partial X_{j}}$$ (7)

$$Q_{i}=A_{ij}^{21}E_{j}+A_{ij}^{22}\frac{\partial T}{\partial X_{j}}.$$ (8)

However, in order to apply Onsager’s theorem to the above coefficients we must rewrite the equations in terms of affinities and fluxes [6]. Thus (7) and (8) become

$$-\frac{J_{i}}{e}=B_{ij}^{11}\frac{1}{T}\frac{\partial M}{\partial X_{j}}+B_{ij}^{12}\frac{\partial }{\partial X_{j}}\frac{1}{T}$$ (9)

$$Q_{i}=B_{ij}^{21}\frac{1}{T}\frac{\partial M}{\partial X_{j}}+B_{ij}^{22}\frac{\partial }{\partial X_{j}}\frac{1}{T},$$ (10)

where *e* is the charge of an electron and *μ* is the electrochemical potential. The Onsager theorem states that $B_{ij}^{kl}=B_{ji}^{lk}$ and thereby reduces the number of independent coefficients. Accordingly, the number of the independent coefficients $A_{ij}^{kl}$ is also reduced. Applying the restrictions of crystal symmetry to the coefficients $A_{ij}^{kl}$ it follows that there are two independent elements $A_{11}^{kl}=A_{22}^{kl}$ and $A_{33}^{kl}$.

The absolute thermoelectric power is defined as the change in voltage per unit change in temperature difference at zero current. So setting *J_i_* equal to zero and using the above results we get:

$$E_{i}=-\frac{A_{ii}^{12}}{A_{ii}^{11}}\frac{\partial T}{\partial X_{i}}.$$ (11)

The voltage *V* is given by

$$\begin{array}{l} V_{i}=\int_{x_{1}}^{x_{2}}E_{i}dx_{i}=-\int_{x_{1}}^{x_{2}}\frac{A_{ii}^{12}}{A_{ii}^{11}}\frac{\partial T}{\partial X_{i}}dx_{i}\\ \;=-\int_{T_{1}}^{T_{2}}\frac{A_{ii}^{12}}{A_{ii}^{11}}dT_{i}. \end{array}$$ (12)

The absolute thermoelectric power is

$$\frac{dV_{i}}{dT}=-\frac{A_{ii}^{12}}{A_{ii}^{11}}=S_{i}.$$ (13)

It follows that we have two possible values of the thermoelectric power, $-\frac{A_{11}^{12}}{A_{11}^{11}}$ and $-\frac{A_{33}^{12}}{A_{33}^{11}}$, corresponding to the one and three axes respectively. We see also that the Onsager theorem does not give us any limitation on the possible numbers of values of the thermoelectric power but only gives us relations between it and other thermoelectric coefficients.

## 6. Thermoelectric Power on the Basis of Simple Ellipsoidal Models

We begin with the Boltzmann transport equation [7]:

$$-\frac{2\pi e}{\hbar }E\cdot \nabla_{k}f+\frac{\partial f}{\partial T}v\cdot \nabla T=\frac{f-f_{0}}{\tau \left(\epsilon \right)}.$$ (14)

Since the electric field and temperature gradient are always small and their squares and products can be neglected, we obtain an approximate solution of eq (14) by putting *f = f*_0_ on the left side where *f*_0_ is the equilibrium distribution. Using

$$\frac{\partial f_{0}}{\partial T}=T\frac{\partial }{\partial T}\left(\frac{\epsilon -\zeta }{T}\right)\frac{\partial f_{0}}{\partial_{\epsilon }}$$

we get

$$f=f_{0}+\tau v_{j}\frac{\partial f_{0}}{\partial E}\left(\frac{\partial T}{\partial X_{j}}\frac{\epsilon }{T}+T\frac{\partial T}{\partial X_{j}}\frac{\partial }{\partial T}\left(\frac{\zeta }{T}\right)+eE_{j}\right),$$ (15)

where *ζ* is the Fermi energy, and *ϵ* the energy. The electric current density is given by [7]:

$$J=\frac{-e}{4\pi^{3}}\int vfdk.$$ (16)

Using our solution for *f* and writing in terms of components of *J* we get:

$$J_{i}=K_{ij}\left[e^{2}E_{j}+eT\frac{\partial }{\partial X_{j}}\left(\frac{\zeta }{T}\right)\right]+L_{ij}\left[\frac{e}{T}\frac{\partial T}{\partial X_{j}}\right],$$ (17)

where

$$-\frac{1}{4\pi }\int_{0}^{\infty }\tau v_{i}v_{j}\frac{\partial f_{0}}{\partial_{\epsilon }}dk=K_{ij}$$ (18)

$$-\frac{1}{4\pi^{3}}\int_{0}^{\infty }\tau v_{i}v_{j}\frac{\partial f_{0}}{\partial_{\epsilon }}Edk=L_{ij}.$$ (19)

Setting *J_i_* = 0, we calculate the thermoelectric power in the same manner as section 5. The result is:

$$S_{i}=-\frac{1}{e}\left[\frac{L_{ii}-\zeta K_{ii}}{K_{ii}}\right]\frac{1}{T}.$$ (20)

(Here we anticipated the results *K_ij_=L*_ij_=0 if *i≠j.*) Assuming a single ellipsoidal energy surface and using $dk=\frac{d\epsilon dS}{{\text{grad}}_{k}\epsilon }$ as the element of volume where *dS* is an element of surface of the ellipsoid, we find upon performing the indicated integration:

$$\begin{array}{l} K_{ii}=\frac{\sigma_{ii}\left(p\right)}{e^{2}}\\ L_{ii}=\frac{kT}{e^{2}}\left(\frac{5}{2}-p\right)\sigma_{ii}\left(p\right). \end{array}$$ (21)

We have again assumed *τ = τ*_0_*ϵ^−p^.* (The above results are obvious on comparison with the expression for *σ_ii_* by Abeles and Meiboom [3].) The thermoelectric power for a single ellipsoid may be written:

$$S_{i}=\frac{-1}{eT}\left[\frac{\left(\frac{5}{2}-p\right)kT\sigma_{ii}-\zeta \sigma_{ii}}{\sigma_{ii}}\right].$$ (22)

The case for many ellipsoids is easily gotten from the above result. Since conductivities simply add we have the result:

$$S_{i}=-\frac{1}{eT}\frac{\left[\left(\frac{5}{2}-p\right)kT\sum_{k}\sigma_{ii}^{\left(k\right)}-\sum_{k}\zeta^{\left(k\right)}\sigma_{ii}^{\left(k\right)}\right]}{\sum_{k}\sigma_{ii}^{\left(k\right)}},$$ (23)

where *k* refers to the *k*th ellipsoid. We note that if *ζ*, measured from the bottom of the ellipsoid, is constant for all ellipsoids of the configuration then the thermoelectric power is independent of direction and is given by

$$S=-\frac{1}{eT}\left[\left(\frac{5}{2}-p\right)kT-\zeta \right].$$ (24)

Thus the simplest case which could yield an anisotropic thermoelectric power and also have tetragonal symmetry would be the five ellipsoid case discussed earlier.

Using the relationship

$$e^{\zeta /kT}=\frac{1}{2}nh^{3}{\left(2\pi mkT\right)}^{-3/2}$$ (25)

we can express the thermoelectric power in terms of the density of electrons, temperature, and effective masses.

### 6.1. Single-Ellipsoid Case

The masses are those indicated in figure 1. Using eqs (24) and (25) we get for the case of a single ellipsoid:

$$S_{i}=S=-\frac{1}{e}G,$$ (26)

where

$$G=2k-k\;\log\;n+\frac{3}{2}k\;\log\;T-k\;\log\frac{1}{2}h^{3}{\left(2\pi mk\right)}^{-3/2}$$ (27)

and

$$m={\left(m_{1}m_{2}m_{2}\right)}^{1/3}.$$ (28)

### 6.2. Four Ellipsoid Case, Figure 2

Carrying out a similar calculation for the four ellipsoid case we get:

$$G=2k-k\;\log\frac{n}{4}+\frac{3}{4}k\;\log\;T-k\;\log\frac{1}{2}h^{3}{\left(2\pi mk\right)}^{-3/2},$$

with eqs (26) and (28) still true. Note that the only change is the division of the density of electrons by 4. This is just what we would expect since equal voltage sources in parallel produce a voltage equal to one of the sources. Thus the presence of many ellipsoids is seen as an effective reduction of the number of carriers.

### 6.3. Five Ellipsoid Models

We shall consider the simplest anisotropic case of five ellipsoids as shown in figure 3. Using eqs (23) and (25), we obtain for the two values of the thermoelectric power:

$$\begin{array}{l} G_{a}=\frac{\frac{n_{1}}{2}\left(\frac{1}{m_{1}}+\frac{1}{m_{2}}\right)G_{1}+\frac{n_{2}}{m_{4}}G_{2}}{\frac{n_{1}}{2}\left(\frac{1}{m_{1}}+\frac{1}{m_{2}}\right)+\frac{n_{2}}{m_{4}}}\\ G_{c}=\frac{\frac{n_{1}}{m_{3}}G_{1}+\frac{n_{2}}{m_{5}}G_{2}}{\frac{n_{1}}{m_{3}}+\frac{n_{2}}{m_{5}}}, \end{array}$$

where

$$\begin{array}{l} G^{1}=2k-k\;\log\frac{n_{1}}{4}+\frac{3}{2}k\;\log\;T-k\;\log\frac{1}{2}h^{3}{\left(2\pi m{\;}^{1}k\right)}^{-3/2}\\ G^{2}=2k-k\;\log\;n_{2}+\frac{3}{2}k\;\log\;T-k\;\log\frac{1}{2}h^{3}{\left(2\pi m{\;}^{2}k\right)}^{-3/2}\\ m^{1}={\left(m_{1}m_{2}m_{3}\right)}^{1/3}\\ m^{2}={\left(m_{4}^{2}m_{5}\right)}^{1/3}. \end{array}$$

Here $S_{a}=-\frac{G_{a}}{e}$ is the thermoelectric power perpendicular to the 4-fold axis and $-\frac{G_{c}}{e}$ is the thermoelectric power along the 4-fold axis.

## 7. Phonon-Drag Contribution to the Thermoelectric Power

In all the former work, the phonon-drag contribution [8, 9] to the thermoelectric power was ignored. According to Frederikse [9] the thermoelectric power is given by:

$$S=-\frac{1}{e}\left[\frac{K_{2}}{K_{1}T}-\frac{\zeta }{T}+\frac{K^{1}}{K_{1}}\right],$$ (29)

where $-\frac{1}{e}\frac{K^{1}}{K_{1}}$ is the extra term due to phonon drag. For the case of lattice scattering (*p* = ½), i.e., $\Lambda_{e_{i}}\gg \Lambda_{e\varphi }$,

$$\frac{K^{1}}{K_{1}}=\frac{3}{4}\pi^{1/2}\frac{\Lambda_{ph}}{\Lambda_{e\varphi }}{\left(\frac{2ms^{2}}{kT}\right)}^{1/2}k,$$ (30)

where

*s*: = speed of sound,
Λ*_ph_*: = phonon-phonon mean free path,
Λ*_eφ_*: = electron-phonon mean free path,
Λ*_ei_*: = electron-impurity ion mean free path,
*m*: = (*m*_1_*m*_2_*m*_3_)^1/3^.

We have the result that the thermoelectric power of a single ellipsoid is given by:

$$S=-\frac{1}{e}\left[2k-\frac{\zeta }{T}+\frac{3}{4}\pi^{1/2}\frac{\Lambda_{ph}}{\Lambda_{e\varphi }}{\left(\frac{2ms^{2}}{kT}\right)}^{1/2}k\right].$$ (31)

Any easy extension to the case of many ellipsoids is obtained by use of [10]

$$S_{i}\underset{\begin{array}{c} \text{total}\\ S_{i} \end{array}}{=}\frac{\sum_{k}S_{k}\sigma_{i}^{k}}{\sum_{k}\sigma_{i}^{k}}.$$ (32)

Here *S_k_* indicates the thermoelectric power of a single ellipsoid and $\sigma_{i}^{k}$ its conductivity in the *i*th direction. This is easily seen to be equivalent to our former method of treating the case of many ellipsoids.

## Figures and Tables

**Table 1:** Zero order magneto-conductive coefficients

Asterisks denote the coefficients which will be taken as the independent quantities. Indices 1, 2, 3 refer to crystal axes, 3 being the 4-fold axis.
$\sigma_{11}^{*}=\sigma_{22}$
$\sigma_{33}^{*}$
All others vanish.

**Table 2:** First order magneto-conductive coefficients

	$\sigma_{123}^{*}=-\sigma_{213}$
	$\sigma_{132}^{*}=\sigma_{321}=-\sigma_{312}=-\sigma_{231}$
All others vanish.	

**Table 3:** Second order magneto-conductive coefficients

	$\sigma_{1111}^{*}=\sigma_{2222}$
	$\sigma_{3333}^{*}$
	$\sigma_{1122}^{*}=\sigma_{2211}$
	$\sigma_{1212}^{*}=\sigma_{1221}\;\sigma_{2121}=\sigma_{2112}$
	$\sigma_{1133}^{*}=\sigma_{2233}$
	$\sigma_{1313}^{*}=\sigma_{2323}=\sigma_{2332}=\sigma_{3232}=\sigma_{3223}=\sigma_{1331}=\sigma_{3131}=\sigma_{3113}$
	$\sigma_{3311}^{*}=\sigma_{3322}$
All others vanish.	

**Table 4:** Magnetoresistive coefficients in terms of magnetoconductive coefficients Expressions are correct to second order in the magnetic field

$\rho_{ii}=\frac{1}{\sigma_{ii}}\;i=1,2,3$
$\rho_{ijk}=-\frac{\sigma_{ij}{\;}_{k}}{\sigma_{ii}\sigma_{jj}}\;i,j,k=1,2,3$
$\rho_{iiii}=-\frac{\sigma_{iiii}}{\sigma \underset{\begin{array}{cc} i & i \end{array}}{2}}\;i=1,2,3$
$\rho_{ijij}=\frac{\sigma_{ikj}\sigma_{kji}}{2\sigma_{ii}\sigma_{jj}\sigma_{kk}}=\frac{\sigma_{ijij}}{\sigma_{ii}\sigma_{jj}}\;\begin{array}{c} i,\;j=1,2,3\\ i\neq j\neq k\neq i \end{array}$
$\rho_{iijj}=\frac{\sigma_{ikj}\sigma_{kij}}{\sigma_{ii}^{2}\sigma_{kk}}-\frac{\sigma_{iijj}}{\sigma_{ii}^{2}}\;\begin{array}{c} i,\;j=1,2,3\\ i\neq j\neq k\neq i \end{array}$

**Table 5:** Configurations for measurement of Hall effect in tetragonal crystals

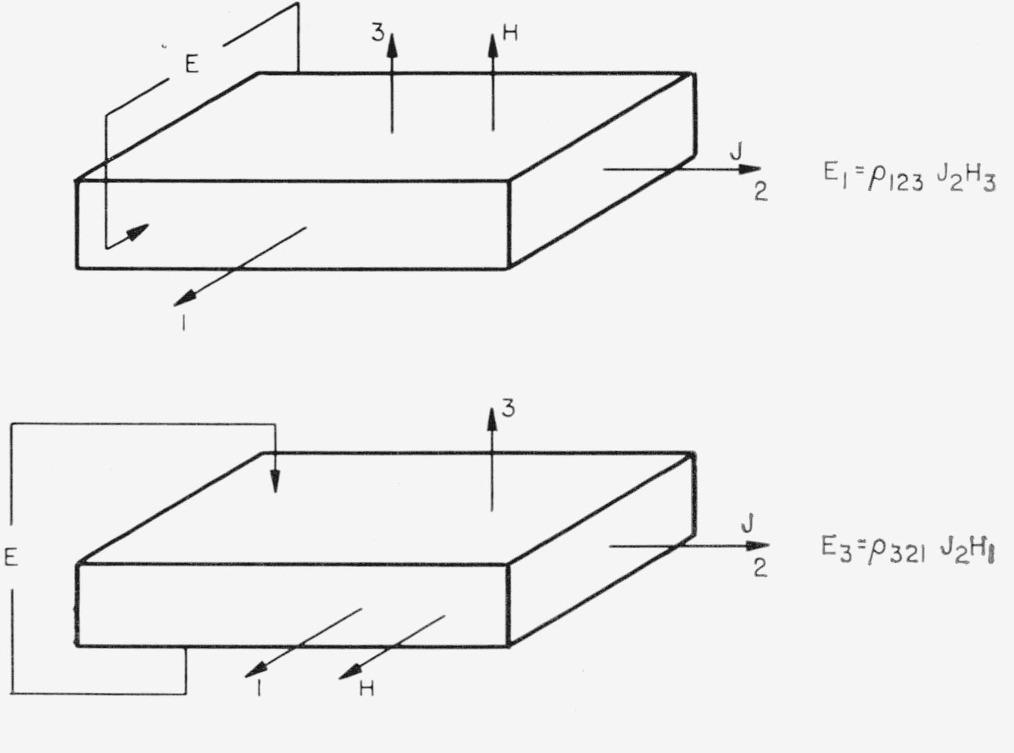

**Table 6:** Configurations for measurement of magnetoresistive coefficients of tetragonal crystals

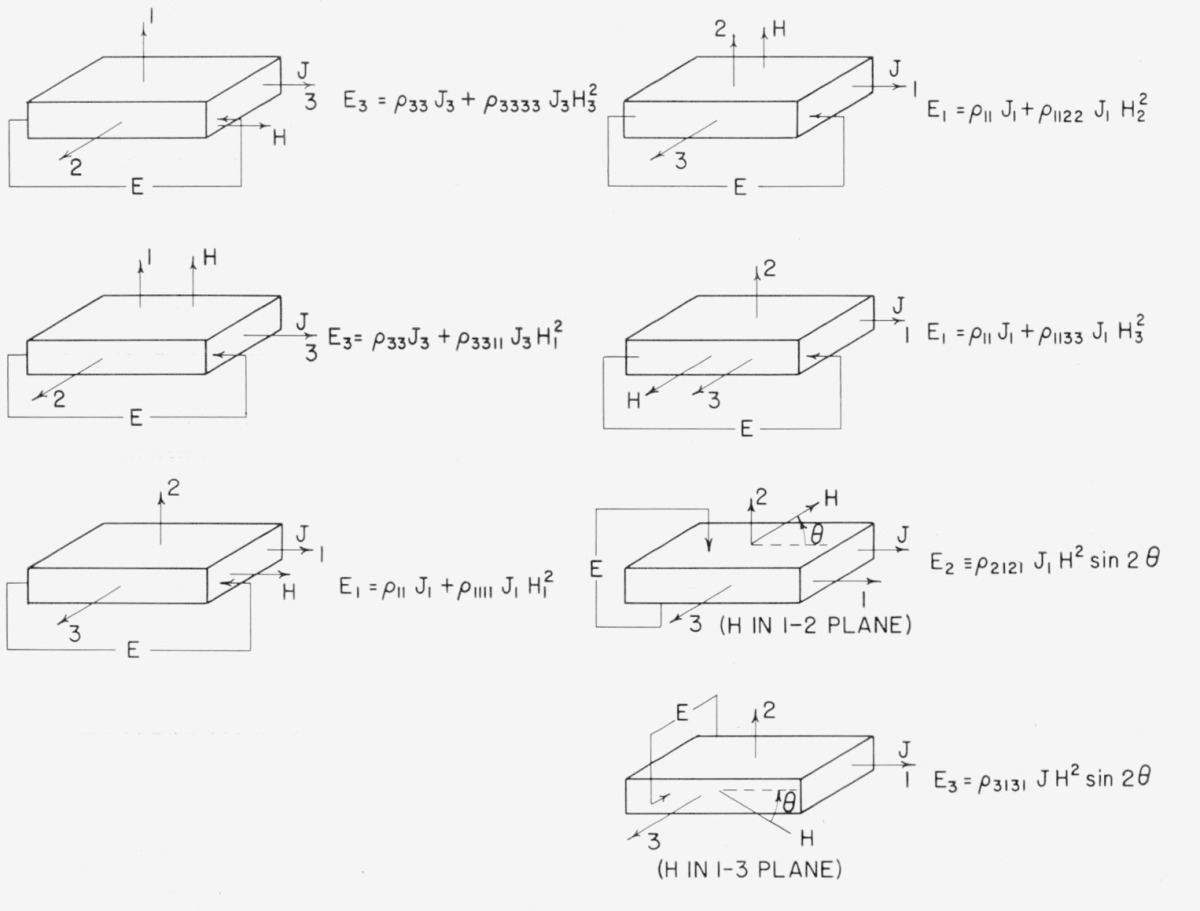

## Footnotes

1 Figures in brackets indicate the literature references at the end of this paper.
